# Supplementary material for: The use of complementary and alternative medicine among hypertensive and type 2 diabetic patients in Western Jamaica: A mixed methods study
Source: PLoS One. 2021 Feb 8;16(2):e0245163. doi: 10.1371/journal.pone.0245163 (PMC7870151; doi:10.1371/journal.pone.0245163)
Supplement: S1 File — (PDF) [file pone.0245163.s002.pdf]

Title: Common alternative therapies used by persons with Chronic Non-Communicable Diseases (Hypertension and Type 2 Diabetes) in Western Jamaica

Interview for Diabetes

ID: ___________ **Date: _________**

1. Are you currently using any alternative treatments/home remedies for your diabetes?

   Yes No

   If yes, which alternative treatment method do you currently use? **Mark all that apply**

   Herbal medicine Exercise

   Nutritional supplements Spiritual healing

   Diet modifications Relaxation techniques

   Manual techniques Other, please specify_______________

2. If you are not currently using an alternative treatment, have you used alternative treatments previously?

   Yes No

   If yes, why did you discontinue use of alternative treatments?

Demographic Information:

3. Parish: Hanover St. James Westmoreland Trelawny
4. District: ______________________
5. Sex: Male Female
6. Date of Birth: ___/___/_____ Age: ______
7. What is the highest level of education you have completed?

   Some Primary Completed Secondary Technical/Vocational

   Completed Primary College/University

   Some Secondary No education

8. Religious Affiliation:

   Catholic Jehovah Witness

   Protestant Rastafarian

   Muslim Adventist

   Other, please specify: ________________

9. What is your marital status?

   Single Widowed/ Divorced

   Married/ Common Law

10. What is your living arrangement?

    House/Apartment/Condo you own

    House/Apartment/Condo you rent

    Stay with family/friend

    Other, please specify: ________________

11. What is your occupation?

None

Professional (e.g. teacher, policeman, doctor, ………..)

Skilled (e.g. plumber, mechanic, electrician, hairdresser, lab technicians ………..)

Clerical (e.g. secretary,

Unskilled (e.g. sanitation/janitorial, ward assistants, porters……..)

Other, please specify_____________________

12. What is your individual monthly income?

No income (being supported by the family)

Less than J$24,800 (min. wage J$6,200 weekly)

J$24,801-J$60,000

J$60,001-J$120,000

J$120,001—J$200,000

11a. Other Income: J$_______________

13. How many people live in the same house with you?

Number of adults (≥18 years) including you: ________

Number of children (under 18 years): ________

Number of children in school: ________

Type 2 Diabetes Mellitus Questions

14. When were you diagnosed with diabetes?

Within the past year

2-5 years ago

6-10 years ago

>10 years ago

15. Are you currently on medication from the clinic or a doctor for your diabetes?

Yes No

16. Where do you receive medications for your diabetes?

Health center

Pharmacy

Hospital

17. Do you have difficulty in picking up your diabetes medicine?

Yes No

If Yes, what difficulty do you experience?

18. Do you get your diabetes medication free of cost or do you have to pay? If you pay, how much?

19. Do you always refill your diabetes medication on-time (i.e. before you run out)?

Yes No

20. If no, why don't you always fill your diabetes medicine on time?

21. Do you always take your diabetes medicine(s) as prescribed?

Yes No

If no, why not?

22. Do you think that your diabetes medicine is controlling your diabetes?

Yes No

If no, why do you think so?

23. Do you experience any side effects from your diabetes medicine(s)?

Yes No

If yes, what side effects do you experience?

Alternative Treatment Use and Knowledge

24. Name the herbal medicines that you take for diabetes and how often you take them.

1. ____________________________________

1-3 days a week	4-6 days a week	7 days a week	Every other week
Once a month	Every 2 months	Every 3 months or less	

2. ____________________________________

1-3 days a week	4-6 days a week	7 days a week	Every other week
Once a month	Every 2 months	Every 3 months or less	

3. ____________________________________

1-3 days a week	4-6 days a week	7 days a week	Every other week
Once a month	Every 2 months	Every 3 months or less	

4. ____________________________________

1-3 days a week	4-6 days a week	7 days a week	Every other week
Once a month	Every 2 months	Every 3 months or less	

5. ____________________________________

1-3 days a week	4-6 days a week	7 days a week	Every other week
Once a month	Every 2 months	Every 3 months or less	

25. Do you think these herbal medicines control your diabetes?

If yes, why do you think so?

26. When you use herbal medicine, do you still take your prescription medication as prescribed?

Yes No

If no, what do you do?

27. Have you received information about alternative treatments?

Yes No

If Yes, what have you heard about alternative treatments for diabetes?

Where or from whom did you receive the information?

28. Have you discussed alternative treatments for diabetes with your healthcare provider?

Yes No

If no, why not?

29. Why do you choose to use alternative medication to treat your diabetes?

30. When do you take alternative medications?

Do you take them when you cannot afford your prescribed medications?

31. Do you experience any negative side effects when taking alternative medication?

If so, what do you experience?

32. Are there any possible harmful effects of using both herbal and prescription medicines at the same time? If so, what are possible harmful effects?

Knowledge Regarding Alternative Treatments

Do you believe:

33. Alternative treatments should **always** be used instead of prescription medication?

Yes No

34. Alternative treatments are **more** effective at treating diabetes than prescription medication?

Yes No

35. If you are experiencing unpleasant side effects of prescription medication, is it okay to stop taking the medicine without consulting your healthcare provider?

Yes No

36. Do you think it is okay to use both prescription medication and alternative treatments at the same time to treat diabetes?

Yes No

37. Do you think that you should always discuss any alternative treatments for your condition with your healthcare provider?

Yes No

Why or why not?

Patient Clinical Data

1. Date diagnosed with Type 2 Diabetes (mm/dd/yyyy) : ___MM___DD_____YYYY

2. Glucose and AIC readings

   Date: ___MM___DD______YYYY Reading: ______________________

   Date: ___MM___DD______YYYY Reading: ______________________

   Date: ___MM___DD______YYYY Reading: ______________________

3. Weight: ___________ Date weighed: ___MM___DD______YYYY
4. Height: ___________ Date measured: ___MM___DD______YYYY

Interview for Hypertension

38. Are you currently using any alternative treatments/home remedies for your hypertension/high blood pressure?

Yes No

If Yes, which alternative treatment method do you currently use?

Herbal medicine Exercise

Nutritional supplements Spiritual healing

Diet modifications Relaxation techniques

Manual techniques Other, please specify: _________________

39. If you are not currently using an alternative treatment, have you used alternative treatments previously?

Yes No

If yes, why did you discontinue use of alternative treatments?

40. When were you diagnosed with high blood pressure?

Within the past year

2-5 years ago

5-10 years ago

>10 years ago

41. Are you currently on medication from the clinic or a doctor for your high blood pressure?

Yes No

42. Where do you receive your medications for your High blood pressure?

Health center

Pharmacy

Hospital

43. Do you have difficulty in picking up your blood pressure medicine?

Yes No

If Yes, what difficulty do you experience?

44. Do you get your blood pressure medication free of cost or do you have to pay? If you pay, how much?

45. Do you always refill your high blood pressure medication on-time?

Yes No

46. If no, why don't you always fill your blood pressure medicine on time?

47. Do you take your blood pressure medicine(s) as prescribed?

Yes No

If no, why not?

48. Do you think that your blood pressure medicine is controlling your high blood pressure?

Yes No

If no, why do you think so?

49. If you get a normal blood pressure reading, do you stop taking your prescription medicine?

Yes No

50. Does a normal (120/80) blood pressure reading mean that:

You are cured

Your blood pressure is normal at the time but you still have high blood pressure

Other, please specify

51. Do you experience any side effects from your high blood pressure medicine(s)?

Yes No

If yes, what side effects do you experience?

Alternative Treatment Use and Knowledge

52. Name the herbal medicines that you take for high blood pressure and how often you take them.

1. ______________________________________

1-3 days a week 4-6 days a week 7 days a week Every other week

Once a month Every 2 months Every 3 months or less

2. ______________________________________

1-3 days a week 4-6 days a week 7 days a week Every other week

Once a month Every 2 months Every 3 months or less

3. ______________________________________

1-3 days a week 4-6 days a week 7 days a week Every other week

Once a month Every 2 months Every 3 months or less

4. ____

1-3 days a week   4-6 days a week   7 days a week   Every other week

Once a month   Every 2 months   Every 3 months or less

5. ____

1-3 days a week   4-6 days a week   7 days a week   Every other week

Once a month   Every 2 months   Every 3 months or less

53. Do you think these herbal medicines control your high blood pressure?

If yes, why do you think so?

54. When you use herbal medicine, do you still take your medication from the doctor as prescribed?

Yes   No

If no, what do you do?

55. Have you received information about alternative treatments?

Yes   No

If Yes, what have you heard about alternative treatments for hypertension?

Where or from whom did you receive the information?

56. Have you discussed alternative treatments for hypertension with your healthcare provider?

Yes   No

If no, why not?

57. Why do you choose to use alternative medication to treat your hypertension?

58. When do you take alternative medications?

Do you take them when you cannot afford your prescribed medications?

59. Do you experience any negative side effects when taking alternative medication?

If so, what do you experience?

60. Are there any possible harmful effects of using both herbal and prescription medicines at the same time? If so, what are possible harmful effects?

Knowledge Regarding Alternative Treatments

Do you believe:

61. Alternative treatments should **always** be used instead of prescription medication?

Yes   No

62. Alternative treatments are **more** effective at treating hypertension than prescription medication?

Yes   No

63. If you are experiencing unpleasant side effects of prescription medication, is it okay to stop taking the medicine without consulting your healthcare provider?

Yes No

64. Do you think it is okay to use both prescription medication and alternative treatments at the same time to treat hypertension?

Yes No

65. Do you think you should always discuss any alternative treatments for your condition with your healthcare provider?

Yes No

Why or why not?

Patient Clinical Data

5. Date diagnosed with HTN: ___MM___DD_____YYYY

6. Blood Pressure readings

Date: ___MM___DD______YYYY Reading: ______________________

Date: ___MM___DD______YYYY Reading: ______________________

Date: ___MM___DD______YYYY Reading: ______________________

7. Weight: ___________ Date weighed: ___MM___DD______YYYY

8. Height: ___________ Date measured: ___MM___DD______YYYY
