# Supplementary material for: The use of complementary and alternative medicine among hypertensive and type 2 diabetic patients in Western Jamaica: A mixed methods study
Source: PLoS One. 2021 Feb 8;16(2):e0245163. doi: 10.1371/journal.pone.0245163 (PMC7870151; doi:10.1371/journal.pone.0245163)
Supplement: S2 File — (DOCX) [file pone.0245163.s003.docx]

***Common herbal therapies used by persons with Chronic Non-Communicable Diseases (Hypertension and Type 2 Diabetes) in Western Jamaica (UAB Public Health/Biomedical Research Training Program)***

MODERATOR’S GUIDE FOR FOCUS GROUP SESSIONS

1. **Introduction of Purpose**

- Thank individuals for their time and for agreeing to participate. Note the purpose of this focus group is to gain an understanding common herbal therapies used by persons with chronic non-communicable diseases.
- Emphasize the value of understanding their experience and encourage full participation.

1. **Introduction of Moderator (Self Introduction)**
2. **Ground Rules/Setup**

- Your participation is voluntary
- We will meet for about 2 hours and then give you your transportation fare
- We will be taping the focus group session
- What you say will be used to help us better understand your circumstances
- There are no right or wrong answers
- Points of agreement and disagreement are welcome

**We ask that you**

- Be direct and honest
- Respect the opinion of others
- Ask questions of me and others
- Be willing to summarize when asked to do so
- Speak loudly enough to be heard
- Use this time to let others really know how you feel
- Allow me to sometimes change the topic

1. **My role as a moderator is to make sure that we hear as many opinions as possible within the time we are allowed and to ask clarifying questions**
2. **Take participants’ questions about the process**
3. **How many of you have participated in a focus group before?**

**Questions**

| 1. Are you currently using any herbal treatments/home remedies for hypertension/high blood pressure or diabetes?  If Yes, which herbs do you currently use for hypertension?  Which herbs do you currently use for diabetes? |
| --- |
| 2. When do you take alternative medications?  (Do you take them when you cannot afford your prescribed medications?) |
| 3. When you use herbal medicine, do you still take your prescription medication as prescribed? |
| 4. If you are not currently using any herbal treatment, have you used herbal treatments previously?  If yes, for which condition and why did you discontinue use of herbal treatments? |
| 5. Have you received information about herbal treatments?  If yes, where/ from whom have you received information?  What have you heard about herbal treatments from others? |
| 6. Have you discussed herbal treatments with your healthcare provider? |
| 7. Do you think herbal treatments should be used instead of prescription medication?  If yes, why? |
| 8. Do you think herbal treatments are as effective at treating the disease as prescription medication? |
| 9. Do you think it is ok to stop taking the prescription medicine if you are experiencing unpleasant side effects from it without consulting a healthcare provider? |
| 10. Do you think it is acceptable to use both prescription medication and herbal treatments simultaneously to treat hypertension/diabetes? |
| 11. Why do you choose to use alternative medication to treat your hypertension/diabetes? |
| 12. Do you experience any negative side effects when taking alternative medication? |
| 13. Are there any possible harmful effects of using both herbal and prescription medicines at the same time?  If so, what are possible harmful effects? |
| 14. Do you think you should always discuss any herbal treatments for your condition with your healthcare provider?  Why or why not? |

**Moderator summarizes the key points of the discussion**

- Did I correctly describe what has been said?

**Ending question**

- Is there anything that we have missed?
- Is there anything that you did not get a chance to say?

1. **Closing**

- Thank all participants
- Have them applaud each other
