# Supplementary material for: The use of complementary and alternative medicine among hypertensive and type 2 diabetic patients in Western Jamaica: A mixed methods study
Source: PLoS One. 2021 Feb 8;16(2):e0245163. doi: 10.1371/journal.pone.0245163 (PMC7870151; doi:10.1371/journal.pone.0245163)
Supplement: Attachment — Submitted filename: PONE-D-20-21696_reviewer.pdf [file pone.0245163.s004.pdf]

PLOS ONE

The Use of Complementary and Alternative Medicine among Hypertensive and Type 2 Diabetic Patients in Western Jamaica: A Mixed Methods Study

--Manuscript Draft--

**Manuscript Number:**	PONE-D-20-21696
**Article Type:**	Research Article
**Full Title:**	The Use of Complementary and Alternative Medicine among Hypertensive and Type 2 Diabetic Patients in Western Jamaica: A Mixed Methods Study
**Short Title:**	CAM use for hypertension and type 2 diabetes mellitus
**Corresponding Author:**	Pauline Jolly, PhD, MPH<br>University of Alabama at Birmingham<br>Birmingham, Alabama UNITED STATES
**Keywords:**	alternative medicine; hypertension; type 2 diabetes mellitus; jamaica
**Abstract:**	Background: Hypertension and type 2 diabetes mellitus are among the top ten causes of death among Jamaicans. The simultaneous use of alternative treatments and prescription medications for these conditions is common in Jamaica. Objectives : To identify beliefs and practices associated with the use of alternative treatments for hypertension and type 2 diabetes mellitus among patients. Methods: A mixed methods study including an investigator- administered survey and focus group discussion sessions was conducted among patients ≥18 years. The quantitative data was summarized and a thematic analysis procedure was conducted to analyze the qualitative data using NVivo. Results : Participants in the study were mostly women (75%) and most (87-90%) were on prescription medication for their condition. Of survey participants, 69% reported taking their medication as prescribed and 70% felt their prescription medicine was controlling their condition. Almost all (98%) reported using CAM, mainly herbal medications, and most (73-80%) felt that herbal medicines controlled their conditions; one-third believed that CAM is the most effective form of treatment and should always be used instead of prescription medication. However, most participants (85.1%) did not believe that prescription medication and CAM should be used simultaneously. Only 10% -24% of participants reported discussing CAM with their healthcare providers. Four themes emerged from the focus group sessions: 1) Simultaneous use of herbal and prescription medicine was perceived to be harmful, 2) Patients did not divulge their use of herbal medicine to healthcare providers, 3) Alternative medicines were perceived to be highly effective, and 4) Religiosity and family elders played key roles in herbal use. Conclusions: This study showed that CAM, mainly herbal medicines, is widely used by patients who have strong beliefs that alternative treatments control their disease. Training healthcare providers in CAM so that they can appropriately advise patients will improve managements of chronic diseases among patients.
**Order of Authors:**	Omolade Adeniyi
	LaTimberly Washington
	Sarah Franklin
	Maung Aung
	Soumya Niranjan
	Pauline Jolly, PhD, MPH
**Additional Information:**	
**Question**	**Response**
**Financial Disclosure**<br><br>Enter a financial disclosure statement that describes the sources of funding for the	This study was supported by the Minority Health International Research Training (MHIRT) grant no. T37-MD001448 from the National Institute on Minority Health and Health Disparities, National Institutes of Health (NIH), Bethesda, Maryland, USA, and the Western Regional Health Authority, Ministry of Health, Jamaica.

work included in this submission. Review the submission guidelines for detailed requirements. View published research articles from *PLOS ONE* for specific examples.<br><br>This statement is required for submission and **will appear in the published article** if the submission is accepted. Please make sure it is accurate.<br><br>**Unfunded studies**<br>Enter: *The author(s) received no specific funding for this work.*<br><br>**Funded studies**<br>Enter a statement with the following details:<br>• Initials of the authors who received each award<br>• Grant numbers awarded to each author<br>• The full name of each funder<br>• URL of each funder website<br>• Did the sponsors or funders play any role in the study design, data collection and analysis, decision to publish, or preparation of the manuscript?<br>• **NO** - Include this sentence at the end of your statement: *The funders had no role in study design, data collection and analysis, decision to publish, or preparation of the manuscript.*<br>• **YES** - Specify the role(s) played.<br><br>* typeset	
**Competing Interests**<br><br>Use the instructions below to enter a competing interest statement for this submission. On behalf of all authors, disclose any competing interests that could be perceived to bias this work—acknowledging all financial support and any other relevant financial or non-financial competing interests.<br><br>This statement **will appear in the published article** if the submission is accepted. Please make sure it is accurate. View published research articles from *PLOS ONE* for specific examples.	The authors have declared that no competing interests exist.

Powered by Editorial Manager® and ProduXion Manager® from Aries Systems Corporation

**NO authors have competing interests**<br><br>Enter: *The authors have declared that no competing interests exist.*<br><br>**Authors with competing interests**<br><br>Enter competing interest details beginning with this statement:<br><br>*I have read the journal's policy and the authors of this manuscript have the following competing interests: [insert competing interests here]*<br><br>* typeset	
**Ethics Statement**<br><br>Enter an ethics statement for this submission. This statement is required if the study involved:<br><br>• Human participants<br>• Human specimens or tissue<br>• Vertebrate animals or cephalopods<br>• Vertebrate embryos or tissues<br>• Field research<br><br>Write "N/A" if the submission does not require an ethics statement.<br><br>General guidance is provided below. Consult the submission guidelines for detailed instructions. **Make sure that all information entered here is included in the Methods section of the manuscript.**	The study was approved by the Institutional Review Board at the University of Alabama at Birmingham and by the Western Regional Health Authority; protocol approval #IRB-170310006.

Powered by Editorial Manager® and ProduXion Manager® from Aries Systems Corporation

**Format for specific study types**<br><br>**Human Subject Research (involving human participants and/or tissue)**<br>• Give the name of the institutional review board or ethics committee that approved the study<br>• Include the approval number and/or a statement indicating approval of this research<br>• Indicate the form of consent obtained (written/oral) or the reason that consent was not obtained (e.g. the data were analyzed anonymously)<br><br>**Animal Research (involving vertebrate animals, embryos or tissues)**<br>• Provide the name of the Institutional Animal Care and Use Committee (IACUC) or other relevant ethics board that reviewed the study protocol, and indicate whether they approved this research or granted a formal waiver of ethical approval<br>• Include an approval number if one was obtained<br>• If the study involved *non-human primates*, add *additional details* about animal welfare and steps taken to ameliorate suffering<br>• If anesthesia, euthanasia, or any kind of animal sacrifice is part of the study, include briefly which substances and/or methods were applied<br><br>**Field Research**<br><br>Include the following details if this study involves the collection of plant, animal, or other materials from a natural setting:<br>• Field permit number<br>• Name of the institution or relevant body that granted permission	
**Data Availability**<br><br>Authors are required to make all data underlying the findings described fully available, without restriction, and from the time of publication. PLOS allows rare exceptions to address legal and ethical concerns. See the PLOS Data Policy and FAQ for detailed information.	No - some restrictions will apply

A Data Availability Statement describing where the data can be found is required at submission. Your answers to this question constitute the Data Availability Statement and **will be published in the article**, if accepted.<br><br>**Important:** Stating 'data available on request from the author' is not sufficient. If your data are only available upon request, select 'No' for the first question and explain your exceptional situation in the text box.<br><br>Do the authors confirm that all data underlying the findings described in their manuscript are fully available without restriction?	
**Describe where the data may be found in full sentences. If you are copying our sample text, replace any instances of XXX with the appropriate details.**<br><br>• If the data are **held or will be held in a public repository**, include URLs, accession numbers or DOIs. If this information will only be available after acceptance, indicate this by ticking the box below. For example: *All XXX files are available from the XXX database (accession number(s) XXX, XXX.*).<br>• If the data are all contained **within the manuscript and/or Supporting Information files**, enter the following: *All relevant data are within the manuscript and its Supporting Information files.*<br>• If neither of these applies but you are able to provide **details of access elsewhere**, with or without limitations, please do so. For example:<br><br>*Data cannot be shared publicly because of [XXX]. Data are available from the XXX Institutional Data Access / Ethics Committee (contact via XXX) for researchers who meet the criteria for access to confidential data.*<br><br>*The data underlying the results presented in the study are available from (include the name of the third party*	Data cannot be shared publicly because it pertains to individuals' health information. Data are available from the corresponding author for researchers who meet the criteria for access to confidential data.

and contact information or URL).<br>• This text is appropriate if the data are owned by a third party and authors do not have permission to share the data.<br><br>* typeset	
Additional data availability information:	

Manuscript

Click here to access/download;Manuscript;FINAL CAM manuscript for PLOSOne.docx

The Use of Complementary and Alternative Medicine among Hypertensive and Type 2 Diabetic Patients in Western Jamaica: A Mixed Methods Study

Short title: CAM use for hypertension and type 2 diabetes mellitus

Omolade Adeniyi, BS[1]; LaTimberly Washington, MS[1]; Sarah Franklin, BS[1]; Maung Aung, MD, MPH[2]; Soumya Niranjan, B Pharm., MS, Ph.D[3]; and Pauline E. Jolly, PhD, MPH[1].

[1]Department of Epidemiology, School of Public Health, University of Alabama at Birmingham, Birmingham, Alabama, USA

[2]Epidemiology Unit, Western Regional Health Authority, Ministry of Health, Montego Bay, St. James, Jamaica

[3]Health Services Administration, School of Health Professions, University of Alabama at Birmingham, Alabama, USA

Corresponding Author:

Pauline E. Jolly; PhD, MPH

Professor

Department of Epidemiology

University of Alabama at Birmingham, School of Public Health

1665 University Boulevard, Birmingham, AL 35294-0022

Telephone: (205)-974-1823

Email: jollyp@uab.edu

Abstract

Background: Hypertension and type 2 diabetes mellitus are among the top ten causes of death among Jamaicans. The simultaneous use of alternative treatments and prescription medications for these conditions is common in Jamaica. **Objectives**: To identify beliefs and practices associated with the use of alternative treatments for hypertension and type 2 diabetes mellitus among patients. **Methods:** A mixed methods study including an investigator-administered survey and focus group discussion sessions was conducted among patients ≥18 years. The quantitative data was summarized and a thematic analysis procedure was conducted to analyze the qualitative data using NVivo. **Results**: Participants in the study were mostly women (75%) and most (87-90%) were on prescription medication for their condition. Of survey participants, 69% reported taking their medication as prescribed and 70% felt their prescription medicine was controlling their condition. Almost all (98%) reported using CAM, mainly herbal medications, and most (73-80%) felt that herbal medicines controlled their conditions; one-third believed that CAM is the most effective form of treatment and should always be used instead of prescription medication. However, most participants (85.1%) did not believe that prescription medication and CAM should be used simultaneously. Only 10% -24% of participants reported discussing CAM with their healthcare providers. Four themes emerged from the focus group sessions: 1) Simultaneous use of herbal and prescription medicine was perceived to be harmful, 2) Patients did not divulge their use of herbal medicine to healthcare providers, 3) Alternative medicines were perceived to be highly effective, and 4) Religiosity and family elders played key roles in herbal use. **Conclusions:** This study showed that CAM, mainly herbal medicines, is widely used by patients who have strong beliefs that alternative treatments control their disease.

Training healthcare providers in CAM so that they can appropriately advise patients will improve managements of chronic diseases among patients.

Introduction

The World Health Organization (WHO) reports that Type 2 Diabetes Mellitus (T2DM) and Hypertension (HTN) are two of the top ten causes of death among the Jamaican population [1]. Worldwide, the prevalence of diabetes has increased significantly since 1995 with over 48 million people currently living with the disease. In Jamaica, it is projected that there will be an additional 33,000 people diagnosed with diabetes by the year 2030, with the current prevalence being at over 200,000 cases [2]. Jamaica's population is approximately 2.7 million people, and non-communicable diseases (NCDs) have increased the economic burden due to the working population being the most affected and decreasing productivity [3].

Complementary and alternative medicine (CAM) has been used for centuries across the globe and consist of a diverse subset of therapies such as dietary supplements, botanicals, traditional Chinese medicine, acupuncture, mind-body medicine, and therapeutic massage [1,4,5]. According to the World Health Organization, roughly 80% of the world's population use at least one form of CAM [6]. Reasons for CAM use vary by country and level of conventional healthcare available among the populations [6]. In countries where many individuals lack access to healthcare resources, and with increasing healthcare costs globally, CAM can often provide a more affordable and accessible alternative to conventional medical care [5]. In high income countries such as the United States of America, CAM use deviates from traditional practices and has been adopted from other countries where CAM consumption is within the dominant structure of healthcare. Types of CAM used in the Caribbean are often methods that have been practiced for generations and have deep cultural and/or religious roots [7]. A survey conducted in Jamaica in 2000 examining use of herbal remedies found that 100% of the participants used herbs, this

study examined Jamaicans from both rural and urban settings and of varying socioeconomic groups [8].

Other research suggests that there are significant associations with herbal use and education, gender, religion, and health insurance status among Jamaicans [9]. It has also been shown that the use of herbal medicines in conjunction with prescription medications is common. Jamaicans are likely to use herbal medicine not only for the treatment of HTN and T2DM but also for illnesses such as the common cold, headache, or diarrhea [9]. In Jamaica and the United States, cases of HTN and T2DM are linked to lifestyle practices; however, it is evident that adherence to prescription medications for chronic diseases is lower for those living in the Caribbean compared to the United States [10].

Although use of CAM has been prevalent worldwide for many years and considered effective for many illnesses, there has been a shortage of experimental studies to determine the true effectiveness of CAM as a plausible treatment for illnesses [11]. One review on drug-herbal interactions discussed seven of the most common herbal medicines used, which included St. Johns Worts (*Hypericum perforatum*), Saw Palmetto (*Serenoa repens*), and Ginkgo Biloba [12]. Each of these herbs have been tested in controlled trials and have been shown to produce both adverse and beneficial effects. For example, a series of prescription medications used to treat chronic and acute conditions were tested with Saw Palmetto, and when paired with Warfarin the anticoagulant effect of Saw Palmetto is increased, but when taken with Metronidazole nausea and vomiting were observed [12]. Saw Palmetto taken alone did not cause serious adverse health effects other than headaches and nausea. Preclinical studies aimed to focus on renal and pancreatic function showed positive therapeutic effects after the administration of low-dose Moringa seeds [13]. Furthermore, histological microscopy was also used to confirm the positive

effects. However, this may differ in the human body where Moringa is ingested in uncontrolled amounts [13].

This mixed-methods research study was conducted to provide a deeper understanding of CAM use for HTN and T2DM by Jamaicans patients. With the expected increase in T2DM and HTN, understanding the beliefs and use of alternative treatments is essential for appropriate guidance of patients for proper management of these chronic diseases. We investigated CAM use, beliefs regarding the effectiveness of prescription medication and CAM and discussion of CAM with healthcare providers (HCPs) by patients. The qualitative portion of the study provided an opportunity for open narratives and richer context within our target population.

Material and Methods

Study design, site, and study population

A cross-sectional mixed-methods study was conducted among patients ≥18 years of age attending clinics for HTN and T2DM in the four parishes of western Jamaica (St. James, Westmoreland, Hanover, and Trelawny) under the Western Regional Health Authority (WRHA). Clinic nurses informed patients of the study when they came in for an appointment. Patients who indicated interest to participate in the study were then introduced to the research team. The potential participants were told about the study by the research team in private rooms in the clinic. Signed informed consent was obtained after the patients were given the opportunity to ask questions and were satisfied that they wanted to participate. Patients were asked if they would be able to participate in a Focus Group Discussion (FGD) session that would be arranged for a later date and contact information was taken for those who said they could. Participants in FGD sessions were not allowed to complete the questionnaires.

Inclusion and exclusion criteria

Participants in this study were adults ≥18 years of age who had been diagnosed with HTN and/or T2DM and were attending health clinics located in one of the four parishes under the WRHA. Those who did not meet these inclusion criteria were excluded.

Quantitative and qualitative data collection

Quantitative

Quantitative data on sociodemographic factors (age, education, employment status, income, and residence), CAM use, beliefs on effectiveness of prescription medication and CAM, and communication with HCPs on CAM use were collected using an investigator-administered questionnaire. Questionnaires took approximately 30-45 minutes to complete.

Qualitative

Five FGD sessions (two in St. James and one in each of the other three parishes) were conducted with a total of 25 participants (approximately five in each group). FGD sessions were conducted in vacant conference and exam rooms at the clinics. Each FGD session lasted approximately 75 minutes and was comprised of male and female participants who had HTN, T2DM, or both. Demographic information was obtained for each participant and FGD questions were tailored to generate discussion on when participants chose to use CAM, their perception of the effectiveness of CAM versus prescription medication, whether participants used prescription medication and CAM concurrently, and communication with their HCPs regarding their medicinal practices.

Ethical approval

The study was approved by the Institutional Review Board at the University of Alabama at Birmingham and by the Western Regional Health Authority; protocol approval #IRB-170310006.

Data Analysis

Quantitative data

The questionnaire data were entered into excel and tables showing numbers and percentages are presented and discussed.

Qualitative data

Transcripts were reviewed by three independent coders (S.J.N.; L.W.; O.A.) and coded using QSR International's NVivo 11.4.3 software using line-by-line coding of all responses to the above- mentioned questions, followed by focused coding for directed codes. We utilized constant comparative method to generate themes from the transcribed data [14]. Trustworthiness was achieved through data triangulation and peer debriefing [15]. Themes are presented in a manner that convey understanding of CAM and prescription medication use.

Results

Quantitative results

Demographic characteristics of questionnaire participants

Sixty patients, aged 35-82 years, completed the questionnaire; 37 (61.7%) had HTN only, 10 (16.7%) had T2DM only, and 13 (21.7%) had both diseases (Table 1). Most participants were female, had a secondary education, and had no income or were earning <J$24,000 a month (USD 1 = JD 122; Table 1).

Table 1. Demographic characteristics of survey participants N=60		
	N	*%*
**Sex**		
**Male**	15	25.0
**Female**	45	75.0
**Age**		
**35-49**	12	20.0
**50-64**	28	46.7
**65-79**	18	30.0
**≥80**	2	3.3
**Marital Status**		
**Single**	29	48.3
**Married**	25	41.7
**Divorced/separated/widowed**	6	10.0
**Education**		
**No education**	4	6.7
**Some/completed primary**	20	33.3
**Some/completed secondary**	33	55.0
**College/University**	3	5.0
**Parish**		
**St. James**	12	20.0
**Westmoreland**	19	31.7
**Trelawny**	14	23.3
**Hanover**	15	25.0
**Income***		
**No income**	17	28.8
**<J$24,000**	18	30.5
**J$24,000-J$60,000**	16	27.1
**>J$60,000**	8	13.6

***1 person did not specify**

Practices and beliefs regarding prescription medication use for T2DM and HTN (Table 2)

A majority of the participants had been diagnosed with T2DM or HTN for over 10 years and all but one patient with HTN reported using CAM (Table 2). The most common CAM method used was herbal medicine; this was followed in sequential order by exercise, spiritual

healing, relaxation techniques, and diet modifications. The top five herbs used for T2DM in sequential order were Guinea Hen Weed (*Petiveria alliacea*), Moringa (*Moringa oleifera*), Garlic (*Allium sativum*), Ginger (*Zingiber officinale*) and Tumeric (*Curcuma longa*). For HTN, the top five herbs used in sequential order were Guinea Hen Weed (*Petiveria alliacea*), Garlic (*Allium sativum*), Moringa (*Moringa oleifera*), Lime (*Citrus aurantiifolia*) and Ginger (*Zingiber officinale*).

Table 2. Practices regarding use of prescription medication for type 2 diabetes mellitus (T2DM) and hypertension (HTN) by survey participants

	**T2DM**		**HTN**	
	N	***%***	***N***	***%***
**When were you diagnosed with T2DM/HTN?**				
**Within the past year**	0	0.0	2	5.4
**2-5 years ago**	2	20.0	11	29.7
**5-10 years ago**	1	10.0	6	16.2
**>10 years ago**	7	70.0	18	48.6
**Are you currently using any alternative treatments/home remedies T2DM or HTN?**				
**Yes**	10	100.0	36	97.3
**No**	0	0.0	1	2.7
**Are you currently on medication from the clinic or a doctor for your T2DM/HTN?**				
**Yes**	9	90.0	32	86.5
**No**	1	10.0	5	13.5
**Do you have difficulty in picking up your T2DM/HTN medicine?**				
**Yes**	1	10.0	11	33.3
**No**	8	80.0	20	60.6
**Sometimes**	1	10.0	2	6.1
**Do you get your T2DM/HTN medication free of cost?**				
**Yes**	4	40.0	23	71.9
**No**	5	50.0	9	28.1

Sometimes	1	10.0		
**How much do you pay for your T2DM/HTN medicine?**				
≤J$2000	2	25.0	5	29.4
J$2001-4999	2	25.0	6	35.3
≥J$5000	4	50.0	6	35.3
**Do you always refill your T2DM/HTN medication on-time**				
Yes	9	90.0	23	71.9
No	0	0.0	6	18.8
Sometimes	1	10.0	3	9.4
**Do you always take your T2DM/HTN medicine(s) as prescribed?**				
Yes	6	60.0	22	68.8
No/sometimes	4	40.0	10	31.3
**Do you think that your medicine is controlling your T2DM/HTN?**				
Yes	7	70.0	22	70.9
No	2	20.0	3	9.7
Sometimes	1	10.0	6	19.4
**Do you experience any side effects from your T2DM/HTN medicine(s)?**				
Yes	4	40.0	15	46.9
No	6	60.0	17	53.1
**If you get a normal blood pressure reading, do you stop taking your prescription medicine?**				
Yes	[redacted]		6	18.8
No			26	81.3
**Does a normal (120/80) blood pressure reading mean that:**				
You are cured	[redacted]		1	2.7
Your blood pressure is normal at the time, but you still have HTN			36	97.3

Most participants (90% with T2DM ad 87% with (HTN) were on prescription medication for their condition and most received their medication from a pharmacy at a health center or a private pharmacy (Table 2). The main difficulties participants reported experiencing in picking up their prescriptions were financial difficulty, issues with their government-issued

insurance card, the pharmacy being out of medication, transportation issues, and need for someone to pick up the medication. Forty percent of diabetic patients and 72% of hypertensive patients reported getting their medication free of cost. Most participants reported filling their prescriptions on time; cost and lack of time were the main reasons given for not filling prescriptions on time. Sixty percent of patients with T2DM and 69% with HTN reported taking their medication as prescribed. Side effects, substitution/preference of alternate medication, forgetting to take, and stopping to see if the medication was working were the main reasons given by participants for not taking their medication as prescribed. Seventy percent of participants felt that their prescription medication was controlling their condition; most of those who felt the medication was not controlling their condition said that the medication was not effective enough. Forty percent of patients with T2DM and 47% with HTN said that they experienced side effects from the prescribed medication. The most common side effects reported were itchiness, stomach pains, and increased urination by patients with T2DM, and dizziness, headache, nausea/stomach ache, muscle pain and increased urination by patients with HTN. The majority of patients with HTN knew that a blood pressure reading of 120/80 did not mean that they were cured or that they should stop taking their prescription medication.

Knowledge, attitudes, and practices regarding CAM use for T2DM and HTN (Table 3)

Most participants (80% with T2DM and 73% with HTN) felt that herbal medicines controlled their conditions. Seventy percent of diabetic patients and 42% of hypertensive patients reported that they do not take their prescription medication as prescribed when they take herbal medicines. The reasons given for not taking herbal and prescription medicines simultaneously were: did not want herbal medicine to interfere with prescription medicine, did not want to take too much medicine, preferred to use herbs, wanted to take less prescription medicine, wanted to

see if herbs were more effective, and did not want blood pressure to drop too low. All of the diabetic patients and 84% of hypertensive patients reported that they had received information about CAM, mainly that CAM can help to control T2DM and HTN, can benefit and are good for the body, and can kill cancer cells. A few patients reported that they heard that herbs were not good for the body and can worsen symptoms. Most participants reported that they received information from family, friends, and community members; only small percentages (10% with T2DM and 24% with HTN) reported discussing CAM with their HCPs. The main reasons patients gave for not discussing CAM with HCPs were, HCPs do not ask about CAM, they did not think of discussing CAM with HCPs, and HCPs do not approve of CAM. The top five reasons given by participants in choosing to use CAM were, CAM helps to control blood sugar/blood pressure, others recommended CAM, CAM is used when they do not have their prescription medication, they wanted to try CAM, and CAM is preferred over prescription medication. About one-quarter of participants reported taking CAM once or twice daily and when they are experiencing symptoms or are unable to afford prescription medication. Over 90% of participants reported no negative side effects of CAM, a few reported sleepiness, dizziness, loss of balance and sinus issues. Twenty percent of patients with T2DM and 27% with HTN felt that there were possible harmful effects of taking prescription and CAM simultaneously. Apart from general side effects such as stomachache and headache, a few patients felt that taking prescription and CAM simultaneously could lower blood sugar or blood pressure too much.

Table 3. Knowledge, attitudes, and practices regarding CAM use for type 2 diabetes mellitus (T2DM) and hypertension (HTN)				
	T2DM		HTN	
	N	%	N	%
**Do you think herbal medicines control your T2DM/HTN?**				
**Yes**	8	80.0	27	73.0
**No**	0	0.0	3	8.1
**Sometimes**	2	20.0	7	18.9
**When you use herbal medicine, do you still take your prescription medication as prescribed?**				
**Yes**	7	70.0	19	57.6
**No**	3	30.0	14	42.4
**Have you received information about alternative treatments?**				
**Yes**	10	100.0	31	83.8
**No**	0	0.0	6	16.2
**Where or from whom did you receive the information?**				
**Family/Friends/community members**	6	60.0	26	63.4
**Internet/printed materials/ health book/radio or television doctors**	3	30.0	9	22.0
**Healthcare provider**	1	10.0	5	12.2
**Own research**	[redacted]		1	2.4
**Have you discussed alternative treatments for T2DM/HTN with your healthcare provider?**				
**Yes**	1	10.0	9	24.3
**No**	9	90.0	28	75.7
**Do you take alternate treatments when you cannot afford your prescribed medications?**				
**Yes**	2	20.0	9	25.7
**No**	7	70.0	25	71.4
**Sometimes**	1	10.0	1	2.9
**Do you experience any negative side effects when taking alternative medication?**				
**Yes**	0	0.0	3	8.1
**No**	10	100.0	34	91.9
**Are there any possible harmful effects of using both herbal and prescription medicines at the same time?**				
**Yes**	2	20.0	9	27.3
**No**	8	80.0	20	60.6
**Unsure**	[redacted]		4	12.1

Participants' beliefs regarding the use of prescription and CAM (Table 4)

The responses from a series of questions that evaluated participants' beliefs regarding the use of prescription medication and CAM and using them simultaneously are presented in Table 4. Thirty-four percent of participants believed that CAM should always be used instead of prescription medication and 40% believed that CAM is the most effective form of treatment for their conditions. Seventy-nine percent indicated that they would communicate negative side effects to their HCP before deciding to discontinue their prescription medications. A majority of participants (85.1%) did not believe that it was okay to use prescription and CAM at the same time and 77% believed that they should always discuss CAM use with their HCP.

Table 4. Participants' perceptions/beliefs regarding se of alternative and prescription treatments

	T2DM		HTN		Total	
	N	%	N	%	N	%
**Alternative treatments should always be used instead of prescription medication**						
**Yes**	4	40.0	12	32.4	16	34.0
**No**	4	40.0	16	43.2	20	42.6
**Sometimes/unsure**	2	20.0	9	24.3	11	23.4
**Alternative treatments are more effective at treating T2DM/HTN than prescription medication**						
**Yes**	5	50.0	14	37.8	19	40.4
**No**	3	30.0	19	51.4	22	46.8
**Sometimes/unsure**	2	20.0	4	10.8	6	12.8
**If you are experiencing unpleasant side effects of prescription medication, is it okay to stop taking the medicine without consulting your healthcare provider?**						
**Yes**	1	10.0	9	24.3	10	21.3
**No**	9	90.0	28	75.7	37	78.7
**Do you think it is okay to use both prescription medication and alternative treatments at the same time to treat T2DM/HTN**						
**Yes**	3	30.0	4	10.8	7	14.9
**No**	7	70.0	33	89.2	40	85.1

**Do you think that you should always discuss any alternative treatments for your condition with your healthcare provider**						
**Yes**	7	70.0	29	78.4	36	76.6
**No**	3	30.0	8	21.6	11	23.4

Qualitative results

Four common themes emerged during FGD sessions (Table 5), which revealed the views and beliefs of participants regarding CAM and prescription medication use for chronic disease management.

Table 5. Focus group themes
**1) Simultaneous use of herbal and prescription medicine was perceived to be harmful.**
**2) Patients did not divulge their use of herbal medicine to healthcare providers.**
**3) Alternative medicines were perceived to be highly effective**
**4) Religiosity and family elders played key roles in herbal use.**

Theme #1: Simultaneous use of herbal and prescription medicine was perceived to be harmful

With regard to the simultaneous use of prescription medications with herbal medicines, most respondents indicated that simultaneous use would cause adverse health effects and that both should not be taken concomitantly. Some respondents expressed the consequences of misusing these treatments, which include low blood pressure or low blood glucose and the possibility of losing consciousness. One participant expressed her personal and interpersonal experiences that summarized this theme:

> *I went to the clinic and someone told me to take it [garlic] like pill but don't use it with the medication because it will knock you out because it happened to me once [meaning to the other person]* (Participant 2, Trelawny, Female)

When I used them at the same time it hit me out and sent me to the hospital and made the pressure very low, so I don't use them together. - (Participant 2, Trelawny, Female)

Relating a similar experience, one participant discussed her regimen for taking prescription medication and herbs.

If I could get some herbs to take and see it helping the sugar and the pressure, I will leave the medication, but you cannot just come off it quick because it will physically hurt you. - (Participant 3, Trelawny, Female))

Theme #2: Patients did not divulge their use of herbal medicine to healthcare providers

Most respondents conveyed that they did not feel it was necessary to communicate CAM use with their HCP. There was a fear that the HCP would disagree with the patients' decision to use CAM and advise against what was working for the patients. There were also indications that because herb use is an intricate part of the Caribbean culture, some herbs were used for other conditions, such as headaches or sinuses and without the intention of treating an individual's HTN or T2DM. Therefore, participants did not think to inform their HCP. One participant stated,

I'm not discussing any herbs with the doctor because when you tell him about herbs he says 'nonsense', 'foolishness' so I continue to drink the herb. I put the medication aside for a while, go on the herb, and then back to the medication. - (Participant 3, Trelawny, Female)

On the other hand, the depth of the HCP-patient relationship was also an indicator of whether a respondent felt comfortable with disclosing information about herb use. There were several physicians working at the clinics that were not originally from Jamaica, which influenced the communication a patient received about herb use.

My doctor is a Nigerian that supports both herbs and medication. I would love my Nigerian doctor to give me the herbs because I know he has an herb book and he knows which ones are best so I can get a list. - (Participant 4, Trelawny, Female)

Theme #3: Alternative medicines were perceived to be highly effective.

Most of our participants perceived CAM to be more effective than prescription medications due to the known possible side effects of prescription medication. They also believed that some prescription medication alleviated the chronic disease but caused other complications.

Yes, I believe the herb is coming from the spiritual background the medication the doctor gives is not as effective. I discovered that even the medication slows down your sex organ and the herb uplifts it. - (Participant 23, St. James, Male)

I think so because a lot of the chemical treatment ends up damaging the body so I think more research should be done in the herbal area, a lot of doctors don't support it because they won't be getting money. - (Participant 5, St. James, Male)

One participant conveyed that although she was in favor of CAM, her prescription medication was equally as effective and provided her with rapid results. When the group was asked if they still take their prescription medication as prescribed when using herbal medications, one participant answered:

I live on my medication but I just take it (herb) for tea so I still take my [prescription] medication. - (Participant 16, Westmoreland, Female)

Theme #4: Religiosity and family elders played key roles in herbal use.

When participants were asked about how they were informed of CAM, most responded that their family and community elders influenced them and that they grew up witnessing people in their communities sharing CAM methods to treat a wide variety of illnesses. Also, many expressed listening to radio doctors explain CAM methods for certain conditions.

I learned a lot from my grandmother even when I had my children, she told me what type of herbs to give them. - (Participant 11, Hanover, Female)

I heard from my parents my mother and father they said that the garlic is good for pressure. – (Participant 14, Hanover, Male)

I hear plenty people talk about the garlic plus a doctor I hear over the radio talks about garlic and says it is good. - (Participant 1, Trelawny, Female)

Within each FGD session, participants expressed that religion played a role in their comfort with using CAM for chronic diseases and other illnesses. For instance, two participants responded:

God put herbs on the earth to heal people, but they are not using it because when I take the pill they give me a lot of side effects. - (Participant 3, Trelawny, Female)

Herbal comes from my religion the Seventh-day Adventist, the prophetess, Ellen, tells us that herbs are good for the body - (Participant 23, Hanover, Male)

Discussion

Results from the quantitative and qualitative analysis mirror each other. Responses from our focus groups provided a better understanding of the societal beliefs that individuals have acquired and shed light on why some individuals prefer CAM methods, particularly herbal remedies, to prescription medication.

The survey revealed that a majority of our participants revealed that they chose to use CAM because prescription medications had side effects that included headaches, decreased energy, and stomachaches. This theme was noted in our FGD sessions where our participants stated that CAM's reduced or nonexistent side effects were preferred over side effects from prescription medicines.

Participants also believed that prescription medications were made from herbs; therefore, they believed that herbal remedies are the purest form of medication and best for the body. Studies conducted to understand the perceived risks and benefits of herbal use by participants, report that herbal use is not perceived as being safer than conventional medicines but are viewed as being more "natural" [16].

The majority of participants stated that they do not use herbal medicines and prescription medication together in fear that combined use will decrease their blood pressure or blood glucose to dangerous levels. Among hypertensive patients, garlic and Moringa teas and eating whole or chopped garlic cloves were most popular. Studies suggest that conventional and herbal drug interactions do cause adverse reactions [12]. In the United States, a study revealed that seven of the most common CAMs used, including St. Johns Wort, Saw Palmetto, and Ginkgo Biloba, can interfere with the pharmacokinetics and pharmacodynamics of prescription drugs and pose unknown negative effects in the body [12]. A series of conventional medications used for chronic diseases was tested with Saw Palmetto and the results included positive and adverse reactions [12]. For instance, when Saw Palmetto is paired with Warfarin, the anticoagulant effect is increased, but if taken with Ibuprofen, serious bleeding is evident [12]. Saw Palmetto taken alone has not proven to cause serious adverse health effects aside from headache and nausea. Preclinical studies in rats on the efficacy of low-dose Moringa seeds showed therapeutic effects

in improving renal and pancreatic function [13]. Although these were positive results, this may differ in the human body where Moringa may be ingested in uncontrolled amounts.

Consistent with other studies, our study participants also believed it is important to communicate their use of CAM with their HCPs, although they chose not to disclose CAM use due to fear of disapproval [8]. A study reported that HCPs do not have adequate knowledge of CAM use, and thus are not able to give medical advice to patients who use CAM [17]. This may in part explain HCPs' reluctance in discussing CAM use with patients. Moreover, literature also suggests that HCPs want to understand the use of CAM and the case-based research behind its use [18]. Therefore, changes to the curriculum for medical, nursing, dietetic and pharmacy students, residents, and fellows to include CAM methods may facilitate increased knowledge of the beneficial and harmful effects of CAM. A finding of interest was that nationality and cultural background of HCPs may influence how information on CAM methods is translated to the public. A participant discussed her interactions with a HCP of African nationality who she is convinced has good knowledge of herbs. Studies conducted in Trinidad and Tobago and Jamaica found a higher level of acceptance of CAM use among Trinidadian HCPs in comparison to those from Jamaica; however, HCPs in both countries seemed to lack proper knowledge about herb-drug use interactions which could be contributing to the lack of communication with patients [8].

As previously mentioned, patients were not able to give specific examples of adverse effects associated with simultaneous use of herbal and prescription drugs. Previous research suggests that there should be increased efforts aimed to increase information on the possible harmful effects of concurrent use with prescription drugs [6].

Religion was an important factor in influencing herbal use in this study. Therefore, clergy members could be used in conjunction with HCPs to relay pertinent information regarding CAM use. Recent studies show that clergy members are key components in health promotion [19].

Limitations

This study has limitations that should be considered in interpreting the results. First, the sample size is small and the convenience sampling method used is prone to inherent bias in representation. As such, the sample may not be representative of the study population and the results may not be generalizable to the population studied. Some patients declined to participate in the study due to lack of trust in providing personal health information to the research staff; they feared that the personal information provided be could be used against them. Secondly, the study sample represents only patients who attended the chronic disease clinics in the WRHA; therefore, the results may not be generalizable to patients attending other clinics in the region or in other regions of Jamaica that were not sampled. Additionally, the data were self-reported and as a result might be subject to social desirability bias. Conducting study interviews and FGD sessions within churches or faith-based institutions might have yielded increased numbers of participants in FGD sessions since CAM use is rooted in religious or community customs; this might have allowed us to reach locations where conversations on CAM use often take place.

Conclusions

This study shows that CAM methods such as use of herbs, prayer, diet, and exercise to treat chronic diseases are a part of daily life for many Jamaicans. The findings from the questionnaires and the themes from the FGD sessions provided new information and insight into the perceptions of respondents regarding their propensity towards CAM use. Patients believed based on their own personal experiences, and those of community/church members, that

alternative treatments are effective with far less adverse effects as compared to prescription medicine. Patients did not discuss their use of CAM with HCPs since they felt that most HCPs did not endorse the use of CAM. These findings help to reveal the importance of HCP-initiated conversations about patient use of CAM. Many patients are aware that there could be adverse reactions to the concurrent use of prescription and herbal treatments, but few were able to state these adverse effects specifically. While patients are not able to articulate possible harm in simultaneous use of prescription and alternative treatments, they have developed their own algorithms of use. Thorough explanations of these effects from an HCP is essential. Thus, the findings from this study highlight the importance of including education on CAM including herbal medication in the curricula of HCPs as this knowledge will allow HCPs to be more confident in advising patients on the use of CAM. This will ultimately lead to better treatment plans and management of these chronic diseases.

Acknowledgments

We thank the nurses in the clinics who facilitated the study and the patients who participated.

References

1. World Health Organization. Jamaica. 2018 [Cited 7 July 2020]. [Internet]. Available from: www.who.int/nmh/countries/jam_en.pdf?ua=1.
2. Wild S, Roglic G, Green A, Sicree R, King H. Global Prevalence of Diabetes: Estimates for the year 2000 and projections for 2030. Diabetes Care. 2004 May;27(5):1047-53.
3. Ferguson TS, Tulloch-Reid MK, Wilks RJ. The epidemiology of diabetes mellitus in Jamaica and the Caribbean: a historical review. West Indian Med J. 2010 Jun;59(3):259-64.
4. World Health Organization. Traditional, Complementary and Integrative Medicine. [Cited 7 July 2020]. In: World Health Organization [Internet]. Available from: https://www.who.int/health-topics/traditional-complementary-and-integrative-medicine#tab=tab_1.
5. World Health Organization. WHO Traditional Medicine Strategy 2014-2023. 2013 [cited 2020 July 7]. Available from: https://apps.who.int/iris/handle/10665/92455.
6. Clement YN, Williams AF, Khan K, Bernard T, Bhola S, Fortuné M, et al. A gap between acceptance and knowledge of herbal remedies by physicians: the need for educational intervention. BMC Complement Altern Med. 2005 Nov 18;5:20.
7. Bahall M, Edwards M. Perceptions of complementary and alternative medicine among cardiac patients in South Trinidad: a qualitative study. BMC Complement Altern Med. 2015;15:99.
8. Delgoda R, Younger N, Barrett C, Braithwaite J, Davis D. The Prevalence of Herbs Use in Conjunction with Conventional Medicines in Jamaica. Complement Ther Med. 2010 Feb;18(1):13-20.

9. Picking D, Younger N, Mitchell S, Delgoda R. The prevalence of herbal medicine home use and concomitant use with pharmaceutical medicines in Jamaica. J Ethnopharmacol. 2011 Sep 1;137(1):305-11.
10. Boume PA, McGrowder DA. Health status of patients with self-reported chronic diseases in Jamaica. N Am J Med Sci. 2009 Dec;1(7):356-64.
11. National Center for Complementary and Integrative Health. Complementary, Alternative, or Integrative Health: What's In a Name?" 2018 July [8 July 2020]. [Internet]. National Institutes of Health. Available from: https://www.nccih.nih.gov/health/complementary-alternative-or-integrative-health-whats-in-a-name.
12. Bressler R. Herb-drug interactions. Interactions between saw palmetto and prescription medications. Geriatrics. 2005 Nov;60(11):32, 34.
13. Al-Malki AL, El Rabey HA. The Antidiabetic Effect of Low Doses of Moringa oleifera Lam. Seeds on Streptozotocin Induced Diabetes and Diabetic Nephropathy in Male Rats. BioMed Res Int. 2015 Jan; 2015.
14. Creswell JW, Clark VLP. Designing and conducting mixed methods research. 3rd edition. SAGE Publications, Inc. 2018.
15. Shenton AK. Strategies for ensuring trustworthiness in qualitative research projects. Education for information. 2004;22(2):63-75.
16. Lynch N, Berry D. Differences in perceived risks and benefits of herbal, over-the-counter conventional, and prescribed conventional, medicines, and the implications of this for the safe and effective use of herbal products. Complement Ther Med. 2007 Jun;15(2):84-91.
17. Milden SP, Stokols D. Physicians' attitudes and practices regarding complementary and alternative medicine. Behav Med. 2004 Summer;30(2):73-82.

18. Patel SJ, Kemper KJ, Kitzmiller JP. Physician perspectives on education, training, and implementation of complementary and alternative medicine. Adv Med Educ Pract. 2017 Jul 25;8:499-503.

19. Bopp M, Baruth M, Peterson JA, Webb BL. Leading their flocks to health? Clergy health and the role of clergy in faith-based health promotion interventions. Fam Community Health. 2013 Jul-Sep;36(3):182-92.

Click here to access/download
Supporting Information
Financial Disclosure Statement.docx
