# Supplementary material for: The use of complementary and alternative medicine among hypertensive and type 2 diabetic patients in Western Jamaica: A mixed methods study
Source: PLoS One. 2021 Feb 8;16(2):e0245163. doi: 10.1371/journal.pone.0245163 (PMC7870151; doi:10.1371/journal.pone.0245163)
Supplement: Attachment — Submitted filename: Response to Reviewers.pdf [file pone.0245163.s005.pdf]

November 2, 2020

RE: PONE-D-20-21696
The Use of Complementary and Alternative Medicine among Hypertensive and Type 2 Diabetic Patients in Western Jamaica: A Mixed Methods Study

Dear Editor,

Thank you for sending the comments from the reviewers of our paper submitted to PLOS ONE. We have made the corrections requested and have attached the revised paper with highlights and a clean version for further consideration. We thank the reviewers for their careful review and believe that the changes have significantly improved the quality of the manuscript. This is a point-by-point response detailing the revisions that have been made and highlighted in the manuscript.

Journal Requirements:

1. Please ensure that your manuscript meets PLOS ONE's style requirements, including those for file naming. The PLOS ONE style templates can be found at https://journals.plos.org/plosone/s/file?id=wjVg/PLOSOne_formatting_sample_main_body.pdf and https://journals.plos.org/plosone/s/file?id=ba62/PLOSOne_formatting_sample_title_authors_affiliations.pdf
Response: We have reviewed the style templates to ensure that the manuscript meets PLOS ONE's style requirements, including those for file naming.

2. Please include additional information regarding the interview guide and questionnaire used in the study and ensure that you have provided sufficient details that others could replicate the analyses. For instance, if you developed a questionnaire as part of this study and it is not under a copyright more restrictive than CC-BY, please include a copy, in both the original language and English, as Supporting Information. With regards to the questionnaire, please also provide further details concerning the development and validation of this tool. Please ensure you have discussed how the sample size was determined for the quantitative arm of this study, for example following a sample size calculation.
Response: We have added detailed information regarding development of the focus group interview guide and the questionnaire used in the study on pages 6-7 and have included a copy of each, in English (the original language) as Supporting Information. The quantitative results serve as evidence for hypothesis generation for future studies. Therefore, our participant sample size (N=60) is sufficient.

3. We note that you have indicated that data from this study are available upon request. PLOS only allows data to be available upon request if there are legal or ethical restrictions on sharing data publicly. For information on unacceptable data access restrictions, please see http://journals.plos.org/plosone/s/data-availability#loc-unacceptable-data-access-restrictions. In your revised cover letter, please address the following prompts:
a) If there are ethical or legal restrictions on sharing a de-identified data set, please explain them in detail (e.g., data contain potentially identifying or sensitive patient information) and

who has imposed them (e.g., an ethics committee). Please also provide contact information for a data access committee, ethics committee, or other institutional body to which data requests may be sent.
b) If there are no restrictions, please upload the minimal anonymized data set necessary to replicate your study findings as either Supporting Information files or to a stable, public repository and provide us with the relevant URLs, DOIs, or accession numbers. Please see http://www.bmj.com/content/340/bmj.c181.long for guidelines on how to de-identify and prepare clinical data for publication. For a list of acceptable repositories, please see http://journals.plos.org/plosone/s/data-availability#loc-recommended-repositories. We will update your Data Availability statement on your behalf to reflect the information you provide.
Response: a) b) There are no ethical or legal restrictions on sharing a de-identified data set. The anonymized data set has been uploaded as a Supporting file titled "S1_Dataset.xlsx" with our revised submission

4. Please include captions for your Supporting Information files at the end of your manuscript, and update any in-text citations to match accordingly. Please see our Supporting Information guidelines for more information: http://journals.plos.org/plosone/s/supporting-information.
Response: We have included captions for the Supporting Information files at the end of the manuscript and have updated in-text citations to match accordingly.

Additional Editor Comments:

Thank you for your submission, reviewers have provided comments and I now invite you to respond to these comments. I draw your attention particularly to the comments in relation to the methods and results as addressing these items will strengthen your work. Appropriate inferential statistic should be included in the data analysis.
Response: We have addressed the comments related to the methods and results that strengthen the paper. Appropriate inferential statistics have been included in the data analysis and the new results presented in Tables 1-4. The text of the quantitative results has been revised accordingly.

In addition, please ensure that scientific names are given for all herbs that are mentioned and that capitalization of drug and plant names are appropriate (i.e. capitals for trade names and lower case for common names of plants/herbs).
Response: This has been done.

5. **Review Comments to the Author**

Reviewer #1:

i. I think this research does a good job highlighting the prevalent use of nontraditional medicines in the management of chronic health conditions and the importance of more explicit exploration of usage by patients on the part of health care providers. Where is the data reporting on the different types of CAM being used by the surveyed patients? of course, the focus was obviously on the use of herbal supplements/foods as representative of CAM (there is passing mention of exercise, spiritual healing, relaxation techniques, diet modification).

Response: We agree with the reviewer that the focus of the study was the use of herbal treatments. We do provide information on the sequential order of the different types of CAM used by the patients, i.e. herbal medicine followed by exercise, spiritual healing, relaxation techniques and diet modification on page 10 of the manuscript. We did not conduct further investigation into these different types of CAM in this study.

ii. The paragraph that begins at line 76 seems unnecessary.
Response: We agree with the reviewer and have deleted the paragraph.

Reviewer #2:

i. The manuscript somehow sound, though some information not presented, e.g. sample size, sampling techniques and procedures, and the data may support the conclusion after addressing the comments.
Response: We have added details on the convenience sampling method and procedures used in the study on pages 5-6 of the manuscript. We have explained that the quantitative study was designed to collect data to serve as evidence for hypothesis generation for future studies. Therefore, a sample size of 60 participants was considered sufficient.

ii. Also, too much limitations while some of them are in the Author's control.
Response: We thank the reviewer and have revised the limitations.

iii. The author didn't indicate the statistical analysis program used to analyze the quantitative data.
Response: The statistical analysis method has been added to the paper as stated below. "The questionnaire data for the 60 participants were entered into excel and imported into JMP Pro 14.0 for analysis. Descriptive statistics were used to describe demographic characteristics of participants using mean ± standard deviation for continuous variables and frequency (percentage) for categorical variables. Demographic characteristics between disease groups were compared using a Fisher's Exact test for categorical characteristics, and an analysis of variance for continuous characteristics. The significance level for these comparisons was set at $p \leq 0.05$."

iv. The author is not ready to make the data available freely, some restrictions will apply
Response: We have uploaded the dataset as a supporting file labeled "S1_Dataset.xlsx".

v. The manuscript presented in an intelligible fashion, but some sentences are not clearly explained, so need some improvements in language.
Response: The manuscript has been revised thoroughly to clarify statements and improve the language.

vi. Research ethics are already considered
Response: Thank you.

vii. Other comments found in the manuscript attached to this document.
Response: We have addressed all of the comments in the manuscript.

Thank you for your kind consideration.

Respectfully,

Pauline Jolly, PhD, MPH
Professor,
Director, UAB Minority Health International Research Training Program
Recipient, 2014 Ellen Gregg Ingalls/UAB National Alumni Society Award for Lifetime Achievement in Teaching
2018 Fulbright Specialist Scholar, Institute of Public Health, Ho Chi Minh City, Vietnam
