# Supplementary material for: The use of complementary and alternative medicine among hypertensive and type 2 diabetic patients in Western Jamaica: A mixed methods study
Source: PLoS One. 2021 Feb 8;16(2):e0245163. doi: 10.1371/journal.pone.0245163 (PMC7870151; doi:10.1371/journal.pone.0245163)
Supplement: Attachment — Submitted filename: Response to Reviewers.pdf [file pone.0245163.s006.pdf]

SCHOOL OF PUBLIC HEALTH

December 21, 2020

RE: PONE-D-20-21696
The Use of Complementary and Alternative Medicine among Hypertensive and Type 2 Diabetic Patients in Western Jamaica: A Mixed Methods Study
Jenny Wilkinson, PhD
Academic Editor, PLOS ONE

Dear Dr. Wilkinson,

Thank you for sending the second set of comments from the reviewers of our paper submitted to PLOS ONE. We have made the corrections requested by the academic editor and the reviewer(s) and have attached a highlighted copy of the manuscript that highlights changes made to the original version labeled as 'Revised Manuscript with Track Changes' and an unmarked version of the revised paper labeled 'Manuscript'. This is a point-by-point Response to Reviewers detailing the revisions that have been made and highlighted in the manuscript.
[Note: HTML markup is below. Please do not edit.]

Reviewers' comments:

Reviewer's Responses to Questions
Comments to the Author

1. If the authors have adequately addressed your comments raised in a previous round of review and you feel that this manuscript is now acceptable for publication, you may indicate that here to bypass the “Comments to the Author” section, enter your conflict of interest statement in the “Confidential to Editor” section, and submit your "Accept" recommendation.
Reviewer #1: (No Response)
Reviewer #2: (No Response)

---

2. Is the manuscript technically sound, and do the data support the conclusions?

The manuscript must describe a technically sound piece of scientific research with data that supports the conclusions. Experiments must have been conducted rigorously, with appropriate controls, replication, and sample sizes. The conclusions must be drawn appropriately based on the data presented.
Reviewer #1: Yes
Reviewer #2: No

---

3. Has the statistical analysis been performed appropriately and rigorously?
Reviewer #1: I Don't Know
Reviewer #2: Yes

Department of Epidemiology
220 Ryals Public Health Building
1665 University Boulevard
205.975.7699
Fax 205.934.8665

The University of Alabama at Birmingham
Mailing Address:
RPHB 220
1720 2ND AVE S
BIRMINGHAM AL 35294-0022

4. Have the authors made all data underlying the findings in their manuscript fully available?

The PLOS Data policy requires authors to make all data underlying the findings described in their manuscript fully available without restriction, with rare exception (please refer to the Data Availability Statement in the manuscript PDF file). The data should be provided as part of the manuscript or its supporting information, or deposited to a public repository. For example, in addition to summary statistics, the data points behind means, medians and variance measures should be available. If there are restrictions on publicly sharing data—e.g. participant privacy or use of data from a third party—those must be specified.
Reviewer #1: Yes
Reviewer #2: No

---

5. Is the manuscript presented in an intelligible fashion and written in standard English?

PLOS ONE does not copyedit accepted manuscripts, so the language in submitted articles must be clear, correct, and unambiguous. Any typographical or grammatical errors should be corrected at revision, so please note any specific errors here.
Reviewer #1: Yes
Reviewer #2: No

---

6. Review Comments to the Author

Please use the space provided to explain your answers to the questions above. You may also include additional comments for the author, including concerns about dual publication, research ethics, or publication ethics. (Please upload your review as an attachment if it exceeds 20,000 characters)
Reviewer #1: the sentence beginning line 66 can be rewritten so it is not run-on: "A survey conducted in 2000 examining use of herbal remedies from both urban and rural Jamaicans found that 100% of participants used herbs".
Response: We thank the reviewer and have revised the sentence to read "A survey that examined the use of herbal remedies among rural and urban Jamaicans of varying socioeconomic groups found that 100% of the participants used herbs [8]."

"advice" should be "advise" line 266.
Response: We thank the reviewer and have made this correction.
"Warfarin" should be "warfarin" line 349.
Response: We have made this correction.
i think the statements made in lines 367-368 and 395-397 are incorrect, participants did report experiences with and/or seem to suspect the prospect of synergistic effects of medications and herbs leading to hypotension and hypoglycemia.
Response: We thank the reviewer for this observation and have deleted these statements.
Reviewer #2: Review Comments to the Author
Please use the space provided to explain your answers to the questions above. You may also include additional comments for the author, including concerns about dual publication, research ethics, or publication ethics

i. The manuscript will sound after addressing the comments.
• Sample size of 60 questionnaires and 25 FGD not enough, maybe Author should explain how is attained to that sample size (state the formula employed),
• Sampling techniques and procedures didn't clearly explain
Response: We have revised the explanation in the methods section (page 5) and added references to justify the sample sizes as follows: "An exploratory design was used for the quantitative portion of this study (Creswell, J. W. and V. L. Plano Clark (2018). Prior studies suggest the sample size (N=60) is sufficient since the exploratory nature of the quantitative survey is the first stage of data collection and provides a rationale for defining future hypotheses for other stages of study (Kutner et al. 1999). In the concurrent quantitative-qualitative design, a smaller qualitative data design sample (N=25) was determined as sufficient (Creswell, J. W. and V. L. Plano Clark (2018).
References:

- Kutner JS, Steiner JF, Corbett KK, Jahnigen DW, Barton PL. Information needs in terminal illness. Soc Sci Med. 1999;48(10):1341-1352.
- Creswell JW, Clark VLP. Designing and conducting mixed methods research. 3rd edition. SAGE Publications, Inc. 2018

• Some information presented in the results section while do not find in the table and the author declared that they are not there, so I don't know why presented in the section while are not there.
ii. This work needs more information than what has been presented in the analyzed data, possibly the tool used missed some questions to grasp those information. For instance the author presented some information in the result text while are not found in the table and she/he declared that data not found.

Response: We have added a table (Table 3) to the paper that shows the total number of participants who selected each herb and total numbers stratified by disease group. These data are for reporting only and not for analysis, so no data analysis was conducted and no data were missed (pages 11-12).

iii. State when and for how long did you collect the data
Response: We have added the dates of the study as May to August 2018 to both the abstract and methods (page 4).

iv. The manuscript needs an English native speaker to make it reader friendly and understandable.
Response: We are native English speakers and professionals. We have re-read and revised the paper to make it more understandable.

v. The manuscript lacks novel part.
Response: This is the first mixed-methods study on CAM use by HTN and T2DM patients in western Jamaica and provides the basis for future studies and interventions on alternative treatments. We agree with the reviewer that we did not expressly highlight the novelty of this study. We have revised the conclusion of the abstract to read "This study is novel in that it provides useful insights into perceptions and use of alternative treatments by patients that can be used by HCPs in developing appropriate interventions to encourage

proper use of prescription medicines and CAM resulting in improved management of these chronic diseases" (pages 2-3).

We have also revised the Conclusion section of the paper to highlight that "the findings from this study indicate the need to include salient information on CAM in the professional curricula of HCPs and can be used to develop appropriate interventions to ensure the proper use of prescription medicines and CAM. This should result in improved management of T2DM and HTN among patients" (page 23).

vi. Find other comments on the manuscript.
Response:

---

7. PLOS authors have the option to publish the peer review history of their article (what does this mean?). If published, this will include your full peer review and any attached files.

If you choose "no", your identity will remain anonymous but your review may still be made public.
Do you want your identity to be public for this peer review? For information about this choice, including consent withdrawal, please see our Privacy Policy.
Reviewer #1: No
Reviewer #2: No
[NOTE: If reviewer comments were submitted as an attachment file, they will be attached to this email and accessible via the submission site. Please log into your account, locate the manuscript record, and check for the action link "View Attachments". If this link does not appear, there are no attachment files.]

Thank you for your kind consideration.

Respectfully,

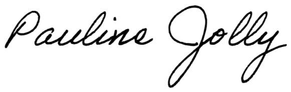

Pauline Jolly, PhD, MPH
Professor,
Director, UAB Minority Health International Research Training Program
Recipient, 2014 Ellen Gregg Ingalls/UAB National Alumni Society Award for Lifetime Achievement in Teaching
2018 Fulbright Specialist Scholar, Institute of Public Health, Ho Chi Minh City, Vietnam
